# Surgical Cases

**Published:** 1854-01

**Authors:** R. D. Mussey

**Affiliations:** Professor of Operative Surgery in the Miami Medical College, at Cincinnati, Ohio


*Surgical Cases. Aneurismal Tumours upon the Ear, successfully treated by the Ligation of both Carotids.* By R. D. MUSSEY, M. D., Professor of Operative Surgery in the Miami Medical College, at Cincinnati, Ohio.

CASE. I. *Aneurismal Tumours upon the Ear treated by Ligation of both Carotids.*—Early in November last, Luther Gordon, æt. 19, accompanied by his physician, Dr. Kramer, came from Indiana, with his head bound up, to this city, on account of aneurismal tumours upon his left ear, and was admitted into St. John's Hospital.

The cavity of the concha was occupied by a pouch which rose above the level of the antitragus, and another covering the tragus and extending some way anterior to it, and pushing outward, was as large as a middling-sized nutmeg. Continuous with the upper part of this was a considerable elevation of the integument which covered the scaphoid fossa, and an inch and a half of the fossa innominata. Below the root of the ear, in the depression between the mastoid process and the ramus of the jaw, and partially covered by the lobulus, was a globular tumour of the same character, as large as a moderate-sized Isabella grape. All these tumours, or pouches, were elastic, and compressible almost to obliteration, pulsated strongly, and seemed to have a communication with each other, like the portions of an arterial varix. The whole circumference of the ear was larger than that of the other, and its integuments everywhere hypertrophied.

L. G. was of a medium stature, with auburn hair and hazel eyes, and, although somewhat delicate in appearance, had enjoyed, from childhood, a pretty uniform health. From birth there was a cutaneous nævus in front of the left ear, but it attracted no particular attention. About eight years ago small elevations of the integument were observed at the points already described as the site of the tumours, in which pulsation was perceptible, especially after exercise. This, together with the size of the tumours, slowly increased, until, a month before he came here, the posterior extremity of the pouch occupying the fossa innominata burst open, causing alarming hemorrhage. This was suppressed by compression; and, subsequently, when the bandage and compresses were removed, the crust covering the opening gave way, and a pulsating jet of arterial blood flowed.

With reference to the treatment of this case, the most promising course which presented itself, was the ligation of one or both carotids. The success which followed the tying of the primitive carotid, by Mr. Travers, in 1809, for "aneurism by anastomosis of the orbit;" and in a similar case by Mr. Dalrymple, in 1813; and also the tying of both carotids, by Dr. J. Mason Warren, in a remarkable case of vascular tumour of the mouth, face, and neck, in 1846, afforded encouragement for this procedure; yet the case I had in 1829, in which I tied both carotids for a large vascular pulsating tumour on the vertex of the head, not having been cured until the tumour was dissected away, left room for doubt whether, in the present instance, the ligature of both carotids even, might not fail of accomplishing the end desired. I determined, however, to resort to the application of a ligature to one of these vessels, possibly to both. The patient had been kept chiefly on farinaceous food since the first outbreak of the hemorrhage, and it was now enjoined upon him to live wholly without animal food until the operation.

On the 18th of November, I tied the left carotid. The pulsation in the tumours ceased on tightening the ligature, and did not afterwards return. His food was strictly farinaceous, with water for his only drink. After the lapse of ten days, a little milk was allowed. No unpleasant symptom occurred, except that when he began to sit up, which he was permitted to do in twelve days, he complained of indistinctness of vision in the left eye. It continued for several days, though less and less marked, till it ultimately subsided altogether. This symptom, indicating a defective supply of blood to the visual apparatus, has been sometimes observed, but I had not myself before noticed it in either of the six cases in which I had applied a ligature to the common carotid. A slow reduction of the tumours took place; but, as it was quite doubtful whether a cure would follow, I proceeded, in four weeks, to ligate the right carotid. A slight effect was observed on the vision of the right eye when the patient began to sit up, similar to what had taken place with the other.

The two operations were performed while the patient was asleep from the inhalation of a mixture of chloroform, one part by measure, and washed sulphuric ether, two parts. Both arteries were tied just below the crossing of the omohyoid muscle. One ligature came away in sixteen days, the other in twenty. After the second operation the reduction in size of the tumours was much more rapid. In about three weeks, collodion was applied and repeated every two or three days. This seemed very much to promote the contraction of the pouches, and on the 28th of January, viz., *seven* weeks from the last operation, L. G. left for home with scarcely a vestige of the tumours remaining. I considered the result of the operations to be a permanent cure.

The last of April, three months after the patient went home, one of his physicians, residing near him, called on me, and gave the assurance that there were no remains of the swelling, and that he regarded the case as perfectly cured.—*Ohio Medical and Surgical Journal.*

